# The NFκB pathway: a therapeutic target in glioblastoma

**DOI:** 10.18632/oncotarget.322

**Published:** 2011-09-05

**Authors:** Lorena Nogueira, Patricia Ruiz-Ontañon, Alfonso Vazquez-Barquero, Francisco Moris, Jose L. Fernandez-Luna

**Affiliations:** ^1^Molecular Genetics Unit, Hospital Valdecilla-IFIMAV, Santander, Spain; ^2^Service of Neurosurgery, Hospital Valdecilla-IFIMAV, Santander, Spain; ^3^Entrechem, Oviedo, Spain

**Keywords:** NFκB, glioblastoma, senescence, IKK inhibitor

## Abstract

Cancer initiating cells have been described to be the only cell population with tumorigenic capacity in glioblastoma multiforme, one of the most aggressive and untreatable cancers. Recent work from our group described that NF&kappa;B pathway was activated in glioblastoma initiating cells undergoing differentiation, and that blockade of this activation promoted senescence of differentiating cells. NF&kappa;B activation in cancer may be the result of either exposure to proinflammatory stimuli in the tumor microenvironment or upregulation of the signaling pathway by upstream regulators. Appropriate control of NF&kappa;B activity, which can be achieved by gene modification or pharmacological strategies, would provide a potential approach for the management of NF&kappa;B related tumors, including glioblastoma. Here, we summarize the current knowledge of the relevance of NF&kappa;B in cancer and its possible role as a target of therapeutic intervention.

## NFκB SIGNALING

NFκB has been associated with many disease states, such as chronic inflammation, cancer, neurodegenerative disorders, diabetes and stroke [1; 2]. NFκB proteins comprise a family of structurally-related transcription factors, including p50 (NFκB1), p65 (RelA), c-Rel, p52 and RelB, all of which have a conserved N-terminal Rel homology domain (RHD) that contains the DNA-binding and dimerization regions. Only p65, RelB and c-Rel contain potent transactivation domains within sequences C-terminal to the RHD. Therefore, p50 and p52 can not act as transcriptional activators by themselves. Dimers of these two proteins have been reported to repress NFκB-dependent transcription in vivo, most likely by competing with other transcriptionally active dimers [3]. NFκB is present in the cytosol in an inactive state, complexed with the inhibitory IκB protein. In the classical pathway, NFκB is regulated by two kinases, IKKα and IKKβ. The latter is particularly important as it phosphorylates IκB, which is subsequently ubiquitinated and degraded by the 26S proteasome, thus leading to the activation of NFκB. Additionally, the IKK complex requires the regulatory IKKγ (NEMO) subunit. The result of these signaling events is the accumulation of the dimeric NFκB transcription factor, containing mainly p50, p65 and c-Rel, in the nucleus where it is able to transactivate different target genes among a large repertoire of more than 200 genes implicated in cell survival/apoptosis, cell growth, immune response, and inflammation. A number of signaling pathways converge on these and other NFκB regulators, providing multiple possibilities for cancer cells to aberrantly activate this trancription factor. Ever-increasing evidence has demonstrated that both the recruitment of NFκB within the nucleus to target genes and the ensuing transcriptional events are actively regulated.

The application of knockout technology to the NFκB pathway has had a major impact on our current understanding of the function of individual components of this signaling cascade. Nearly every technique available for the manipulation of the mouse genome has been employed for the analysis of the NFκB signaling pathway, including the transgenic overexpression of activators or inhibitors of NFκB, conventional and conditional gene knockouts and also specific modifications of endogenous genes through targeted knock-ins [4]. Mice lacking IKKγ or IKKβ die early during embryogenesis with liver degeneration, a phenotype similar to that of p65-deficient animals. The analysis of cells lacking individual IKK subunits led to the identification of two distinct pathways regulating NFκB activation. IKKβ and IKKγ are essential for NFκB activation via the canonical pathway, which is induced by proinflammatory stimuli such as TNFα, IL-1β and lipopolysaccharide (LPS), and leads to the phosphorylation and degradation of IκB proteins and the nuclear accumulation of NFκB. IKKα seems to be dispensable for this process, but it regulates, in a IKKγ- and IKKβ-independent manner, NFκB activation via the alternative pathway. This pathway is activated downstream of receptors controlling lymphoid organogenesis and lymphocyte development, such as the lymphotoxin-β receptor and the B cell activating factor (BAFF) receptor, and induces processing of the NFκB2 (p100) precursor, resulting in nuclear accumulation of mostly p52/RelB dimers [5]. IKKβ and IKKα kinases also display a degree of functional redundancy, exemplified by the presence of residual proinflammatory signal-induced NFκB activity in IKKβ-deficient cells.

## NFκB IN CANCER

NFκB is one of the major transcription factors associated with cancer and it has been implicated in many hallmarks of cancer development, including growth factor-independent proliferation, inhibition of apoptosis, limitless replicative potential and tissue invasion and metastasis [6]. NFκB also seems to play relevant activities in initiating cells. To this end, it has been shown that inhibition of this transcription factor maintains pluripotency of mouse embryonic initiating cells [7], promotes condrogenesis by human mesenchymal initiating cells [8], and controls the number of neural progenitor cells [9; 10]. Some cancers are caused by viruses that encode activators of the NFκB pathway, which block the cell death inherent in viral transformation [11]. In a variety of hematologic and solid tumors, NFκB is constitutively activated by a number of different mechanisms including mutation of upstream components of this pathway in tumor cells [12]. This experimental evidence shows that the NFκB is central to the pathogenesis of many cancer types, providing the basis for the development of strategies to block this pathway. NFκB signaling is activated transiently when normal B lymphocytes respond to antigens, but lymphomas accumulate genetic alterations in a number of NFκB regulators that constitutively activate this pathway. Similarly, normal plasma cells activate NFκB in response to ligands, but their malignant counterparts, multiple myeloma cells, carry a variety of genetic mutations that stabilize NFκB-inducing kinase (NIK), which leads to constitutive activation of NFκB. Whereas the involvement of NFκB activation in hematologic malignancies has been well established, identifying a role for NFκB in solid tumors required the use of mouse models in which tumor induction depends on inflammation, thus mimicking inflammation-driven cancers in humans [13]. The first one was a mouse model for colitis-associated cancer, a type of colon cancer that appears in patients with ulcerative colitis, a chronic inflammatory bowel disease [14]. These authors showed that deletion of IKKβ in intestinal epithelial cells leads to a dramatic decrease in tumor incidence without affecting tumor size, which is linked to increased epithelial apoptosis during tumor promotion. However, deletion of IKKβ in myeloid cells promoted a decrease in tumor size, most likely due to a reduced expression of cytokines that may serve as growth factors for tumor cells. Another inflammation-linked cancer is hepatocellular carcinoma (HCC), the most common form of liver cancer. This cancer mostly develops in the context of chronic viral hepatitis. An animal model of HCC, associated with chronic liver inflammation, is the Mdr2 knockout mouse. This model was used to propose that NFκB constitutes the link between inflammation and cancer [15]. The authors monitored hepatitis and cancer progression in knockout mice, showing that the inflammatory process triggers hepatocyte NFκB through upregulation of TNFα in adjacent endothelial and inflammatory cells. Interestingly, inhibition of NFκB signaling did not affect early phases of hepatocyte transformation, but inducible inhibition in later stages of tumor development resulted in apoptosis of transformed hepatocytes and failure to progress to HCC. However, another mouse model based on the conditional deletion of hepatocyte IKKγ exhibited spontaneous liver damage and developed HCC even without any injection of a carcinogen [16]. Thus, IKKγ was proposed as a tumor suppressor in the liver.

Chronic inflammatory disorders such as systemic lupus erythematosus and rheumatoid arthritis have consistently been associated with the development of B-cell non-Hodgkin lymphoma [17]. NFκB has been shown to be constitutively activated in these disorders and it is considered responsible for the maintenance of chronic inflammation due to cytokines such as TNFα, IL-1, IL-6, and IL-8 [18]. Thus, chronic immune stimulation along with genetic and environmental factors and some immunosuppressive drugs might be involved in lymphomagenesis in these patients.

There are many other tumors that rarely arise in the context of underlying inflammation and yet are dependent on inflammatory processes, mainly as a consequence of tumor progression. This may be exemplified by prostate cancer. Deletion of IKKβ in prostate epithelial cells proved to have no effect on prostate cancer development or progression. However, deletion of IKKβ in hematopoietic-derived cells slowed down the development of androgen-independent prostate cancer and inhibited the appearance of metastases [13], most likely due to reduced levels of NFκB target cytokines that may serve as growth and survival factors for cancer cells. These associations are of great interest as they provide information about mechanisms of tumor development and progression, and may help identify novel therapeutic targets.

## NFκB IN GLIOBLASTOMA

The association between NFκB and glioblastoma (GBM) is known for more than 15 years. A number of authors have described that the NFκB pathway is constitutively activated or is upregulated in response to different stimuli, mainly cytokines, in GBM cells. EGFR gene amplification and overexpression are a particularly striking feature of GBM, observed in 40-50% of tumors. In approximately 50% of GBMs with EGFR amplification, a highly oncogenic EGFR mutant (EGFRvIII) can be detected. This mutant is generated from a deletion of exons 2 to 7 of the EGFR gene, which results in an in-frame deletion of 267 amino acids from the extracellular domain of the receptor. EGFRvIII is unable to bind ligand, and it signals constitutively. Using GBM cells carrying this truncated receptor, it has been shown that EGFR induces association between the docking protein Grb2-associated binder 1 (Gab1) and the tyrosine phosphatase SHP-2. This protein complex appears to be critical for linking EGFR to NFκB transcriptional activity via the PI3-kinase/Akt signaling axis in GBM cells [19]. In line with this, a recent work showed that deletion of IκB has an effect similar to that of EGFR amplification in the pathogenesis of GBM and is associated with comparatively short survival [20]. In this study, 790 human GBM samples were analyzed for genetic alterations of IκB and found that this gene is often deleted but not mutated in GBM and that deletion of IκB and amplification of EGFR showed a pattern of mutual exclusivity. This result strengthen the role of the NFκB signaling pathway in the pathogenesis and/or progression of GBM. Other works have associated activation of NFκB with resistance to different cell death strategies such as TRAIL, a member of the TNF family that selectively induces apoptosis in certain tumor cells, and O6-alkylating agents that cause DNA damage, the standard therapy of GBMs [21; 22]. NFκB activation has also been associated with worse prognosis in GBM. A recent work showed that the receptor interacting protein 1 (RIP1), an upstream activator of the NFκB pathway, negatively regulates p53 through upregulation of mdm2, a well-known inhibitor of this tumor suppressor protein, establishing a mechanistic link between NFκB and p53 [23]. These authors also showed that RIP1 and mdm2 are commonly overexpressed in GBM, but not in low grade gliomas, and that increased expression of RIP1 confers a worse prognosis. A number of NFκB target genes, including cytokines, cell cycle regulators, anti-apoptotic proteins and cell adhesion molecules, have been proposed to influence the invasion capacity and resistance to chemotherapy of GBM cells. All these data support the key role of NFκB in GBM and provide a mechanistic explanation for some of the main features that make this tumor so aggressive and resistant to chemotherapy.

## ACTIVATION OF NFκB IN GLIOBLASTOMA INITIATING CELLS UNDERGOING DIFFERENTIATION

Very recently, we have demonstrated that activation of NFκB is upregulated during differentiation of glioblastoma initiating cells (GICs) [24]. The study revealed the upregulation of cytokines and chemokines, including IL-8, IL-11, IL-6, IL-1β, IL-15, and CCL2 and genes with diverse biological functions, including cell cycle regulation (Cyclin D1), cell adhesion (CD44) and proteolysis (TFPI2, PLAU), which are known targets of the NFκB pathway. This result was consistent with the nuclear localization of the p65 subunit of NFκB (active state) in a high proportion of differentiated cells as compared with the progenitor cell population where more than 90% of the cells contained p65 within the cytoplasm (inactive state). The upregulated activity of NFκB in differentiating GICs was further confirmed by the increase in binding of p50-p65 dimers to a consensus DNA sequence, and a higher level of phosphorylated IκB. It has been shown that expression of NFκB significantly increased following differentiation of embryonic stem (ES) cells with retinoic acid. Additionally, nuclear localization of NFκB in response to TNFα, an agonist of NFκB signaling, was evident in differentiated, but not in undifferentiated ES cells [25]. Similar results have been obtained by other authors [7]. Interestingly, overexpression of NFκB proteins promoted differentiation, whereas inhibition of NFκB signaling increased expression of pluripotency markers [7]. We have observed similar results in a reduced number of GIC cultures treated with TNFα (Nogueira L and Fernandez-Luna JL, personal communication). Following treatment for up to 16h, GIC-containing neurospheres underwent moderate differentiation as assessed by morphological features. Furthermore, normal differentiation was retarded with respect to untreated cells in the presence of a NFκB signaling inhibitor. The low activation of NFκB detected in GICs suggests that NFκB may be dispensable for survival and proliferation of these tumor progenitor cells, which correlates with data showing that p65 immunoreactivity is abundant in the cytoplasm of the majority of cells within neurospheres derived from embryonic mouse brain, but only occasional weak nuclear localization (active state) is detected [9]. Our group also showed that blockade of NFκB in GICs undergoing differentiation by using genetic strategies or small-molecule inhibitors accelerates maturation, promotes proliferation arrest and induces cellular senescence (polyploidy, increase in pH2AX-containing nuclear foci, induction of β-gal, telomere shortening) [24]. There are evidences in other cell systems that suggest the role of NFκB in terminal differentiation. For instance, signaling through the receptor activator of NFκB (RANK) not only promotes proliferation but also inhibits the terminal differentiation of mammary epithelial cells [26]. RANK overexpression in a transgenic mouse model results in increased mammary epithelial cell proliferation during pregnancy and impaired differentiation of lobulo-alveolar structures, suggesting a role for aberrant RANK signaling during breast tumorigenesis. NFκB has also been associated with senescence. In line with this, downregulation of NFκB has been associated with cellular senescence in a number of systems. Treatment of hepatocellular carcinoma cells with rapamycine plus 5-fluorouracil led to senescence accompanied by downregulation of NFκB transcriptional activity [27]. Additionally, it has been described that Tid1, a homologue of the Drosophila tumor suppressor gene l(2)tid, contributes to senescence of rat embryo fibroblasts by acting as a repressor of NFκB signaling [28] and that replicative cellular senescence of human WI-38 fibroblasts was accompanied by a strong decrease in nuclear NFκB [29].

We also showed that knockdown of Cyclin D1, which is consistently upregulated during differentiation of GICs, reproduces part of the phenotype observed by inhibition of the NFκB signaling. In good agreement with these findings, Cyclin D1 knockdown in neuroblasts promoted a reduction in cell proliferation and an extensive neuronal differentiation [30]. We propose that direct activation of the NFκB pathway may be an efficient strategy for differentiating glioblastoma-initiating cells to maintain their proliferative potential, and blockade of this transcriptional pathway drives tumor cells into senescence. Further support to this come from c-Neu/ErbB2-transformed mouse mammary gland cells, cultured as sphere-forming progenitors, which assume typical epithelial appearance following a differentiation stimulus, and grow to some extent, but undergo cellular senescence following inhibition of NFκB signaling [31].

## TARGETING THE NFκB PATHWAY IN CANCER

**Figure 1:**
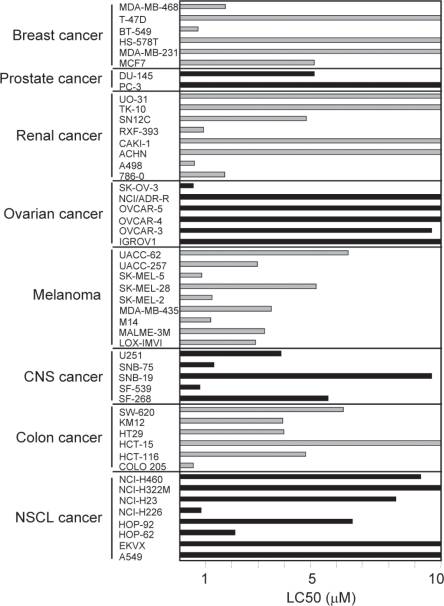
Response of solid tumor-derived cell lines to the IKKβ inhibitor, EC-70124 The small molecule inhibitor was added to 24h old cultures of each of the 50 cell lines used in the panel. After 48h of incubation, cells were fixed and stained with sulforhodamine B, and the total stain quantitated by absorbance determinations. Through the use of a time 0 control, the 50% lethal concentration (LC50) was determined. Those bars that reach the upper limit of the histogram represent LC50 values higher than 100 μM.

Since the identification of the NFκB signaling pathway, many studies demonstrated the association between upregulated activity of NFκB and cancer, mostly by protecting tumor cells from apoptosis. Therefore, this transcriptional pathway participates in the onset or progression of many human cancers. It is considered that the anti-cancer activities of many anti-inflammatory drugs are, at least in part, related to the inhibition of NFκB. In addition, many reseacrh groups and companies have developed novel compounds acting on the NFκB pathway by using a number of systems, including computational models to virtually screen chemical databases and combinatorial biosynthesis. Some of these agents are supposed to be NFκB specific (i.e. IKK inhibitors) while others have wide-range biological activities (i.e. proteasome inhibitors). Given the tight regulation of NFκB by IκB molecules and the central role of IKKβ in phosphorylation and degradation of the inhibitor [32], IKKβ is a very promising target for therapeutic strategies aiming at interfering with NFκB activation. To this end, a number of compounds have been described to inhibit IKKβ with a wide range of inhibition activities (Table 1). Other widely used inhibitors of the NFκB pathway, such as Bay 11-7082 and sulfasalazine, have been shown to inhibit both IKKα and IKKβ [33-35]. Sulfasalazine is being tested in clinical trials for its use in chronic inflammatory disorders, such as rheumatoid arthritis. In addition, a prospective, randomised study of the safety and efficacy of sulfasalazine for the treatment of progressing malignant gliomas was initiated in 2005 but the lack of clinical response and the appearance of side effects led to its early termination [36]. Many of the IKKβ inhibitors identified so far are being used in cultured cells or in animal models and some look promising not only because of their capacity to control inflammation but also because of their potential to be used as antitumor drugs reducing tumor cell survival or inducing sensitisation to chemotherapy. Among these, PS1145, MLN120B (both from Millennium Pharmaceuticals, Cambridge, MA) and BMS-345541 (Bristol-Myers Squibb, Princeton, NJ) have been studied in different tumor models. BMS-345541 sensitises tumor cells derived from mantle cell lymphoma, multiple myeloma, neuroblastoma, melanoma and ovarian cancer to apoptosis induced by therapeutic agents such as TRAIL, melphalan, temozolomide or carboplatin [37-40]. Sensitisation is produced in most cases by reducing the expression of NFκB target genes that belong to the antiapoptotic machinary of the cell (i.e., Bcl-2 and IAP family members). Similarly, PS-1145 has also been shown to be effective at promoting sensitisation of cells from different solid tumors and hematologic malignancies to apoptosis inducers [41; 42]. A novel glycosylated indolocarbazol, EC-7014, has been recently identified as a potent and selective inhibitor of IKKβ [43]. We have demonstrated that this inhibitor is able to promote replication arrest and senescence of glioblastoma initiating cells undergoing differentiation in vitro. Moreover, intravenous treatment of immunodeficient mice bearing human GIC-derived tumors with EC-70124 induced senescence of tumor cells but no ultrastructural alterations of the brain parenchyma were detected [24]. These findings support the rationale for therapeutic strategies aimed to block key factors within the NFκB signaling pathway in GBM cells. Consistently, it has been recently described that pharmacological inhibition of NFκB decreased the viability of GBM cell lines while presented a low toxicity to normal astrocytes, which indicated selectivity to cancer cells [44]. In this case, inhibition was achived with proteasome inhibitors and other compounds that promote an indirect blockade of NFκB. EC-70124 has been tested in a panel of 50 human cell lines derived from 8 different types of solid tumors: breast cancer, prostate cancer, renal cancer, ovarian cancer, melanoma, CNS cancer, colon cancer and lung cancer (Figure 1). The best response was achieved in melanoma followed by central nervous system (CNS) cancer cells, mainly glioma cells (mean LC50 of 3.05 and 4.24 μM respectively) whereas ovarian cancer cells were the most resistant to the inhibitor. NFκB activity has been shown to be enhanced in many cancers, including melanoma, mainly due to deregulations in upstream signaling pathways such as Ras/Raf, PI3-kinase/Akt, and NIK [45]. Proteasome inhibitors, such as PS-341 (bortezomib, velcade), selectively and reversibly inhibit the 26S proteasome and prevent the breakdown of many regulatory proteins through the inhibition of the ubiquitination-proteasome process. One of these proteins whose breakdown is impaired is IκB. This presumably leads to NFκB inactivation and reversal of the malignant phenotype that it regulates. PS-341 inhibits growth of melanoma cells *in vitro* and *in vivo* either alone or in combination with temozolomide [46]. This proteasome inhibitor is already approved for the treatment of patients with relapsed multiple myeloma or mantle cell lymphoma, and a number of clinical trials are underway to determine the value of PS-341 as an effective therapy for malignant melanoma.

**Table 1:** IKKβ small molecule inhibitors

INHIBITOR	*IC_50_	REFERENCE
BMS-345541	0.3 μM	J Biol Chem. 2003;278:1450-6.
IMD-0354	0.28-3.0 μM	Blood. 2005;105:2324-31
TPCA-1	0.018 µM	J Pharmacol Exp Ther. 2005;312:373-81
PS1145	0.088 μM	J Biol Chem. 2005;280:20442-8
MLN120B	0.06-1.0 μM	Blood. 2006;107:4266-73
IKI-1	0.07 μM	Cancer Res. 2008;68:9519-24
KINK-1	2.8-21 μM	J Natl Cancer Inst. 2008;100:862-75
NSC 676914	17 μM	Mol Cancer Ther. 2009;8:571-81
PF-184	0.037 μM	J Pharmacol Exp Ther. 2009;330:377-88
VH01	20.3 μM	BMC Bioinformatics. 2010;Suppl 7:S15
LASSBio-1524	20 μM	Eur J Med Chem. 2011;46:1245-53
* half maximal inhibitory concentration		

Increasing evidence indicates the need of preclinical studies and clinical trials using potent and selective inhibitors of the kinase activity of IKKs to assure the specificity against a key pathway for a number of cancer cell types, including glioblastoma. To this end, there are undergoing clinical trials with novel IKK inhibitors such as SAR113945, a small molecule inhibitor from Sanofi-Aventis that is being evaluated in patients with knee osteoarthritis. This and other compounds that may pass the safety stage, could be adecuate candidates to be studied in cancer patients.

## UNANSWERED QUESTIONS AND FUTURE DIRECTIONS

Increasing evidence support the key role of the NFκB signaling pathway in the pathogenesis and/or progression of GBM. There are many signaling routes that converge in the activation of NFκB but their relevance in GBM is poorly understood. Among these pathways, DNA damage signaling appears to be constitutively activated in gliomas, as documented by a number of markers, mostly activation of ataxia telangiectasia mutated (ATM) kinase. Upon DNA damage, this protein triggers multiple events to promote cell survival and facilitate repair. ATM augments cell survival by activating nuclear factor NFκB. Therefore, further investigation on the association between ATM and NFκB in GBM might expand the targeted therapeutic options to avoid NFκB-dependent tumor cell survival and thus resistance to chemotherapeutic drugs. Aditionally, a detailed study of the vast array of upstream regulators of NFκB in GBM cells is still to come. NFκB is emerging as a potential target for therapeutic intervention in GBM. Although a number of small molecule inhibitors of the NFκB pathway, mainly inhibitors of IKK proteins, are already available, more specific inhibitors of IKKβ and other upstream kinases need to reach clinical studies to prove their efficacy in GBM patients.
